# Colicin E2 Expression in *Lactobacillus brevis* DT24, A Vaginal Probiotic Isolate, against Uropathogenic *Escherichia coli*

**DOI:** 10.1155/2014/869610

**Published:** 2014-02-04

**Authors:** Disha Trivedi, Prasant Kumar Jena, Sriram Seshadri

**Affiliations:** Institute of Science, Nirma University, Sarkhej-Gandhinagar Highway, Chharodi, Ahmedabad, Gujarat 382481, India

## Abstract

Novel therapeutic approaches are needed to combat the urinary tract infection in women. During menstruation elevated protein concentration and increase in oxygen and carbon dioxide concentrations with decrease in vaginal Lactobacilli all together contribute to urinary tract infections. *Lactobacillus* species are a predominant member of the vaginal microflora and are critical in the prevention of a number of urogenital diseases. In order to increase antimicrobial potential of vaginal Lactobacilli, bacteriocin colicin E2 which has specific activity against uropathogenic *Escherichia coli* has been overexpressed in vaginal probiotic *Lactobacillus brevis* DT24. Recombinant *Lactobacillus brevis* DT24 expressing colicin E2 showed much higher inhibitory activity against uropathogenic *Escherichia coli* than wild type *L. brevis* DT24 *in vitro*. Efficacy of probiotic *Lactobacillus brevis* DT24 expressing colicin E2 protein is required for further *in vivo* evaluation.

## 1. Introduction

Urinary tract infection (UTI) is the most widespread infection in women worldwide after intestinal infection [1]. UTIs affect an estimated 1 out of 3 women before the age of 24 [2, 3]. Up to 40 to 50% of the female population will develop a symptomatic UTI at some time during their lives [2, 3] or develop complicated UTIs [4]. Recurrent UTI (rUTI) is a common syndrome in young healthy women. Previous studies suggest that 27% to 44% of women, who experienced an initial UTI, develop rUTI [5, 6].

UTI has the potential for severe and life-threatening sequelae if left untreated or undertreated. Possible sequelae include pyelonephritis which can lead to renal scarring and sepsis [7]. UTI can be particularly dangerous in pregnant women in whom it has been shown that up to 50% of those with asymptomatic bacteriuria (ABU) leads to develop pyelonephritis. In addition, these women experience higher rates of intrauterine growth restriction and low birth weight infants. The presence of a UTI has also been shown to increase the risk of preterm labor, preterm birth, pregnancy-induced hypertension, preeclampsia, amnionitis, and anemia [8].

*Escherichia coli* are among the most significant human pathogens, responsible for up to 90% of all community acquired and almost 50% of nosocomial UTIs.* E. coli *is a ubiquitous human pathogen responsible for both community and hospital-acquired infections [9, 10]. A number of virulence determinants facilitate the ability of uropathogenic *E. coli *to colonize the urinary tract and exert cytopathic effects, including type 1 fimbriae [11], P fimbriae [12], Dr adhesions [13], hemolysin [14, 15] cytotoxic necrotizing factor 1 [16], flagella [17], capsule polysaccharide [18], lipopolysaccharide O antigen [19], and TonB-dependent iron transport systems [20]. During UTI outer membrane proteins of uropathogenic *E. coli *like porins (OmpA, OmpC, OmpX, NmpC, and LamB) and outer membrane assembly factors, including YaeT and YeaF, as well as nucleoside and vitamin B12 receptors Tsx and BtuB, are overexpressed [21].

Colicin E2 (ColE2) is a proteinaceous bacterial toxin produced by some strains of *Escherichia coli *that exhibits inhibitory activity against uropathogenic *E. coli *[22] via binding to an outer membrane receptor—the TonB-dependent vitamin B12 transporter, BtuB [23].

Earlier reports suggest that there is an increased antibiotic resistance in *E. coli*. Initially, resistance was limited to certain specific antibiotics, such as ampicillin or trimethoprim [24], but recently the horizon of resistance has expanded, with the emergence of broad resistance to third generation antibiotics [25–28].

*Lactobacillus *species are an important group of bacteria that inhabit the gastrointestinal tract and represent the predominant microorganism found in the healthy vaginal ecosystem [29–33], producing a variety of compounds [34–38] that inhibit potentially pathogenic microorganisms. It is for this reason that *Lactobacillus *species have been studied as a potential probiotic for the prevention and treatment of urogenital disease in women [39–42]. During menstruation, the vaginal pH becomes neutral, most likely due to the influx of menses blood, which has a pH range of 6.9 to 7.2 [43]. In addition, menses blood in the vagina also results in an elevated protein concentration [43] and increase in oxygen and carbon dioxide concentrations [44], leading to decrease in vaginal Lactobacilli, which all contribute to colonization of uropathogenic *E. coli *which leads to UTIs [45–48].

As a means of increasing the antimicrobial capabilities of *Lactobacillus *species against uropathogenic *E. coli *(UPEC), probiotic *L. brevis *DT24, isolated from vagina of healthy women [49], was used for overexpression of colicin E2.

The objective of this study was to express ColE2 structural gene *ceaB *and immunity gene *ceiB *from *E. coli *into probiotic *Lactobacillus brevis *DT24, secreting colicin E2 to exclude a uropathogenic *E. coli *competitively and completely. In this study genetically engineered probiotic *Lactobacillus brevis *DT24 would exert its antimicrobial effect against the target pathogen directly through the expression of ColE2 gene and indirectly through beneficial properties inherent in probiotics.

## 2. Materials and Methods

### 2.1. Bacterial Strains, Plasmids, and Growth Conditions

Bacterial strains used in this study are listed in Table 1. *Lactobacillus brevis *DT24 (NCBI Accession no. JX163909) was routinely cultured in MRS broth (HiMedia, Mumbai) at 37°C for 48 h. For the analysis of expression of colicin E2, recombinant strains were grown in basal MRS medium supplemented with 2% xylose. *Escherichia coli *DH5*α*, *Escherichia coli *BL21 D3, and *Escherichia coli *NCTC 50133 were routinely cultured in Luria Bertani (LB) broth and agar at 37°C for 16 to 24 h and uropathogenic *Escherichia coli *MTCC 729 (Microbial Type Culture Collection, Chandigarh) was routinely cultured in nutrient broth and agar at 37°C for 16 to 24 h. Chloramphenicol (Cm) and streptomycin (Stpr) were used for the selection of plasmids.

### 2.2. DNA Manipulation, *E. coli* Competent Cell Preparation, Transformation, and PCR

Plasmid DNA was isolated using GeneJet Plasmid Miniprep Kit as per instruction (Fermentas). DNA cloning and transformation procedures followed as previously described [51]. Restriction enzymes were purchased from New England Biolabs. Ligation was carried out by using Rapid Ligation Kit (Fermentas).

### 2.3. Construction of Plasmids and Transformation of *Lactobacillus*

The expression plasmid pSLP111.3, a type of secretion expression vector containing *Slp*A as secretion signal and having a cell wall anchor domain, was kindly gifted by Prof. Jos Seegers (Falco Biotherapeutics, The Netherlands). Nucleic acid manipulation and cloning procedures was performed according to standard procedures [51].

Colicin E2 gene fragment of about 2.01 kb encoding the colicin E2 structural gene (*ceaB*) and colicin E2 immunity gene *ceiB *was obtained from the plasmid pColE2-P9 (*E. coli* genetic resource centre, Yale University, USA) by polymerase chain reaction (PCR) amplification with the primers 5′-GGATCCATGAGCGGTGGCGAT-3′ (forward) containing a *Bam*HI site (underlined) and 5′-CTCGAGTCAGCCCTTTTAAATCCTGA-3′ (reverse) containing an *Xho*I site (underlined). PCR conditions were as follows: 30 cycles of 30 s at 94°C, 30 s at 58°C, and 2 min 20 s at 72°C after denaturing for 4 min at 94°C.

**Figure 1:**
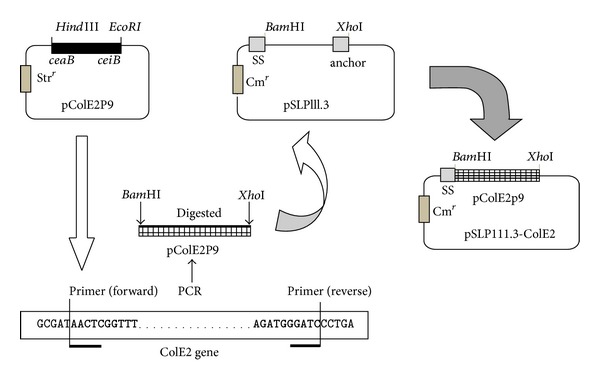
The construct of recombinant vectors expressing colicin E2. The ColE2 gene (*ceaB*) and its immunity gene (*ceiB*) on plasmid DNA (pCoLE2-P9) were amplified using the polymerase chain reaction (PCR) with the primers. The PCR product was cleaved with BamHI and XhoI restriction endonuclease and inserted into the corresponding sites of pSLP111.3, giving rise to pSLP111.3-pCoLE2-P9.

The PCR product of colicin E2 gene was cleaved with *Bam*HI and *Xho*I restriction endonuclease and inserted into the corresponding sites of pPSL111.3 digested by *Bam*HI and *Xho*I, respectively, giving rise to pSLP-ColE2 (Figure 1).

Electroporation of *L. brevis *DT24 was carried out as previously described [52] with some modifications. In brief, a 2% inoculum of an overnight culture was grown in MRS medium supplemented with 1% glycine at 37°C until the OD660 of culture was 0.2 to 0.3. The cells were harvested and washed twice with cold washing buffer (5 mM sodium phosphate pH 7.4 and 1 mM MgCl_2_). The cells were then resuspended to 1% of the original culture volume in a cold electroporation buffer (1 M sucrose, 3 mM MgCl_2_). For electroporation, 45 *μ*L of the cell suspension was mixed with 50 to 500 ng of plasmid DNA and subjected to 2.5 kV, 200-*Ώ*, and 25 *μ*F electric pulse in a 0.2 cm cuvette by using a Genepulser II electroporation system (Bio-Rad Lab). After the pulse, 450 *μ*L of cold MRS was immediately added to the cell suspension, kept on ice for 10 min, and incubated for 3 h at 37°C. The transformants were plated onto MRS agar plates and incubated for 48 to 72 h. The transformation efficiency was calculated as the number of transformants per microgram of plasmid DNA.

### 2.4. Molecular Weight Determination

Transformed ColE2 gene having *Lactobacillus brevis *DT24 was grown in MRS medium at 37°C for 24 hr and centrifuged (10,000 rpm, 30 min, and 4°C) to collect supernatants. The collected supernatants were filter sterilized (0.20 *μ*m; Axiva). Ammonium sulfate was slowly added to the cell-free supernatants to 60% saturation and stirred for 4 h at 4°C and centrifuged (10,000 rpm, 30 min, and 4°C). The precipitate was resuspended in 10 mL of 25 mM ammonium acetate buffer (pH 6.5) and desalted by dialysis using a 1,000 Da cutoff dialysis membrane (Sigma) against the same buffer. Sodium dodecyl sulfate–polyacrylamide gel electrophoresis (SDS-PAGE) was used for further separation, as described by Laemmli [53].

### 2.5. Antimicrobial Activity of Recombinant *L. brevis *DT24 Expressing Colicin E2

Antimicrobial activity of cell-free supernatant (filter sterilized) was measured by well diffusion assay. Filtrates were neutralized (set to pH 6.5) with 5 N NaOH. Nutrient agar plates were flooded with pathogenic bacteria (0.1% of overnight grown uropathogenic *E. coli *strain), air-dried, and then 6 mm diameter wells were punctured in each plate. The prepared supernatants were poured into respective wells (25 *μ*L) and incubated for 24 h at 37°C. *L. brevis *DT24 was used as negative control and *E. coli *NCTC 50133 was used as positive control.

## 3. Results

### 3.1. Construction of an Expression Vector

Plasmid pSLP111.1 is a high-copy-number plasmid which includes a constitutive XylA promoter, secretion signal *Slp*A, cell wall anchoring domain of *Prt*P, and the chloramphenicol resistance gene (*cat *gene).

**Figure 2:**
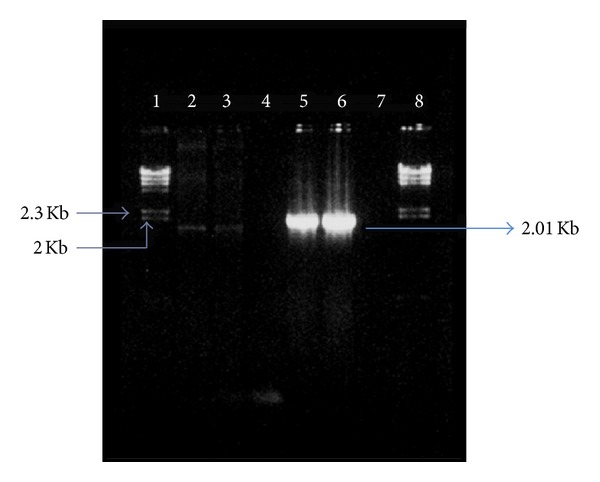
PCR amplification and confirmation of gene colicin E2.

Plasmid pSL-ColE2 was constructed by inserting a 2.01 kb PCR amplicon obtained from pColE2-P9 DNA containing *ceaB *and *ceiB *into pSLP111.1 (Figure 1). Transformants containing pSL-ColE2 demonstrated inhibitory activity against the uropathogenic *E. coli* (Figure 1). The presence and orientation of the *ceaB *and *ceiB *insertion in pSL-ColE2 was verified by PCR (Figure 2).

### 3.2. Extracellular Expression of Colicin E2

**Figure 3:**
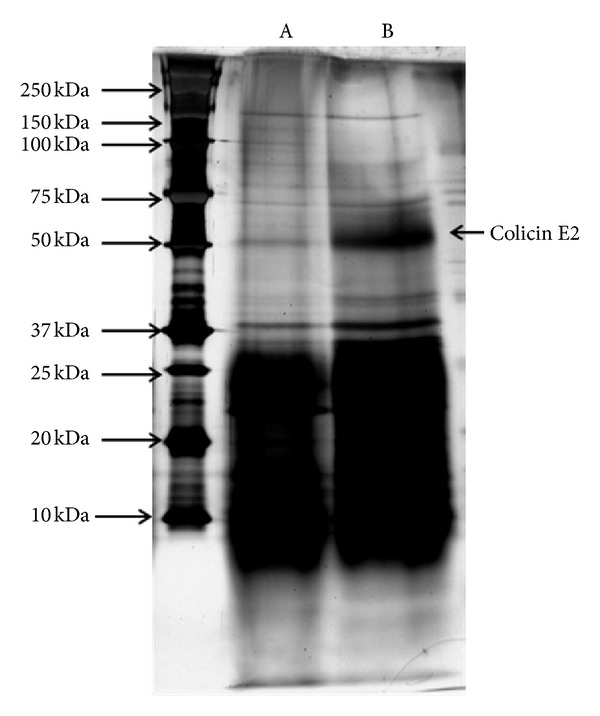
SDS-PAGE of ColE2 protein. (A) Total protein of *L. brevis *DT24 and (B) total protein of *L. brevis *DT24-Col E2. Colicin E2 arrow indicates extracellular secretion of ColE2 protein.

*L. brevis *DT24 transformants harboring recombinant pSL-ColE2 were tested for extracellular expression in 500 mL of MRS broth. Transformant and control strains containing vector only were incubated with vigorous shaking (200 rpm), and cell growth was monitored by checking optical density at 600 nm. After 24 hrs of growth 52 kDa protein was observed in SDS-PAGE analysis (Figure 3).

### 3.3. Antimicrobial Activity of Recombinant *L. brevis* DT24 Expressing Colicin E2

**Figure 4:**
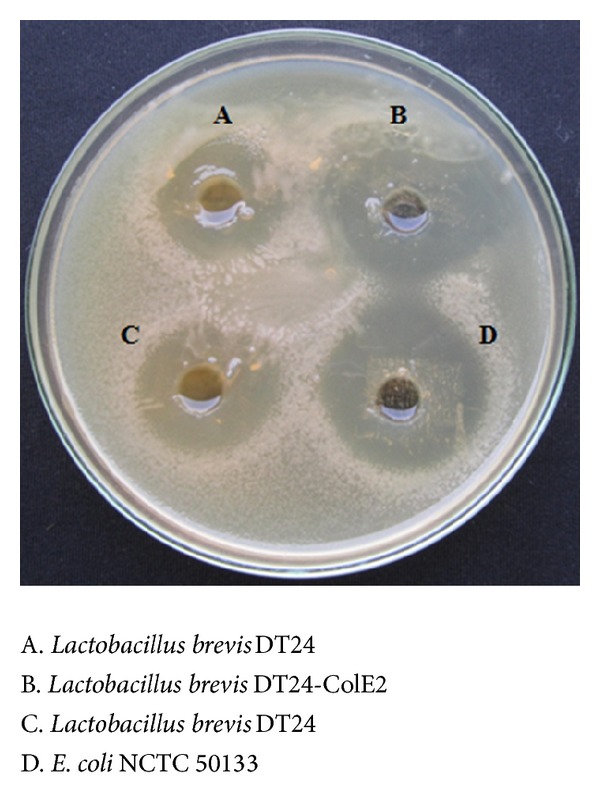
Antimicrobial activity of *L. brevis *DT24-ColE2 cell-free supernatant against uropathogenic *E. coli *as an indicator strain.

Antimicrobial properties of transformed *L. brevis* DT24-ColE2 showed higher zone of inhibition (56 mm) compared to Wild Type *L. brevis* DT24 (23 mm) were shown in Figure 4. But there is no difference in the inhibition zone showed by *L. brevis* DT24 ColE2 and *E. coli* NCTC 50133.

## 4. Discussion 

Various techniques have identified *Lactobacillus *species as the predominant microorganism found in the vaginas of most healthy and fertile women [40, 41]. *Lactobacillus *species have been studied as a potential probiotic for the prevention and treatment of urogenital disease in women [39–42]. During menstruation, the vaginal pH becomes neutral, most likely due to the influx of menses blood, which has a pH range of 6.9 to 7.2 which leads to lower Lactobacilli number in vagina and chances of infections like urinary tract infections and bacterial vaginosis.

Colicin E2 production by a nonpathogenic organism may have clinical applicability as a means to prevent catheter-associated urinary tract infection [22]. Evans et al. [54] demonstrated the possibility of developing oral whole-cell vaccines against diarrhea caused by enterotoxigenic *E.coli *by modifying this *E. coli *by the *in situ* destruction of chromosomal and plasmid DNAs by ColE2. The colicin operon is carried on a plasmid and includes a structural gene (*ceaB*) encoding for the bacteriocin, an immunity gene (*ceiB*) that protects the producer cell from the toxin, and a lysis gene (*celB*) that leads to death of the producer cell and release of ColE2 into the surrounding medium [23]. Sensitivity of gram-negative microorganisms to ColE2 is conferred by the binding of the bacteriocin to an outer membrane receptor, the TonB-dependent vitamin B12 transporter, BtuB [23]. After transport across the membrane, ColE2 acts as an endonuclease, degrading the DNA of the sensitive cell. During UTIs uropathogenic *E. coli *overexpresses surface protein BtuB which can act as receptor for binding colicin E2.

The colicin E2 gene (ColE2) from *E. coli *cloned in shuttle secretion vector (pSLP111.3) and successfully expressed in our lab probiotic isolate *Lactobacillus brevis *DT24 and its impact on the inhibitory activity of host organism were examined. The expression of ColE2 in *Lactobacillus brevis *DT24 was studied by isolating proteins extracellularly.

One of the challenges of transforming ColE2 in *Lactobacillus* is the differences in the transport mechanisms of bacteriocins in gram-negative and gram-positive microorganisms [55]. In gram-negative microorganisms, ColE2 is thought to be released into the surrounding medium after CelB-mediated lysis of the producer cell. Expression of *celB* leads to changes in the cell envelope and results in activation of Omp LA, an outer membrane phospholipase A [23]. Mutation or deletion of the lysis protein has been shown to interfere with release, and in such cases, colicin remains in the cytoplasm [23]. In gram-positive microorganisms, secretion does not occur through cell lysis and is not a lethal event for the cell. Instead, secretion is dependent on a signal peptide, which typically contains conserved double-glycine regions and is mediated by a bacteriocin-specific transport system or the *sec*-dependent export pathway [56]. Although ColE2 does not contain a signal peptide to direct the secretion of the protein, it is predicted to contain 6 double-glycine regions at the N terminus, which may function in a manner analogous to a signal peptide. Thus, ColE2 may be secreted by a gram-positive host without lysis of the producer cell. This feature is important if the genes encoding for colicin production are to be transferred to and expressed by a *Lactobacillus* [57].

This study demonstrated that genes associated with bacteriocin production from a gram-negative microorganism could be cloned, expressed, and secreted by a gram-positive microorganism in the absence of a lysis protein (CelB) and with addition of a signal peptide. In the present work, genes associated with ColE2 production (*ceaB* and *ceiB*) were transferred to *L. brevis* DT24, probiotic isolate from vagina. The level of ColE2 production by the colicin-producing transformants of *L. brevis* DT24 was similar to that of *E. coli* NCTC 50133, from which the ColE2-encoding genes (pColE2-P9) were derived.

Secretion of ColE2 proteins into the surrounding medium by *E. coli* NCTC 50133 and the pSLP111.3-ColE2 transformants occurred before cell leakage was observed. The mechanism proposed for secretion of ColE2 from *E. coli* involves release of the colicin caused by the lysis protein CelB [58–63]. Braun et al. [64] found that inactivation of *celB* resulted in decreased release of colicin from the cells, compared with cells containing intact *celB*.

Cloning and expression of ColE2 in *L. brevis* DT24 allowed evaluation of the transformant as a bioactive compound for use in treatment of UTI. Similar strategies were used for treatment of* Staphylococcus aureus* infection by expressing antimicrobial protein lysostaphin in vaginal probiotic *Lactobacillus plantarum* WCFS1 [65] and inhibition of HIV by expressing anti-HIV proteins which were capable of blocking the HIV entry into human peripheral blood mononuclear cells in probiotic organism *Lactobacillus reuteri* RC-14 [66].

## 5. Conclusion 

This study has demonstrated that the expression of *E. coli* colicin E2 (ColE2) into *Lactobacillus* showed increased expression of colicin E2 in extracellular level to inhibit the infectious disease occurred by uropathogenic *E. coli*. The probiotic properties of host *Lactobacillus brevis* DT24 were increased in the terms of antimicrobial activity against pathogenic *E. coli*. Oral administration of probiotics has clear effects on the numbers and activities of intestinal and fecal bacteria. The administration oral probiotics help to reduce the transfer of yeast and urogenital pathogenic bacteria from the rectum to vagina [67]. It may be possible to use these *L. brevis* DT24-ColE2 probiotics in biotherapy (i.e., as vehicles for the secretion of colicin E2 in the gastrointestinal tracts as well as urovaginal tract for the treatment of UTI as well as other gastrointestinal infectious diseases).

## Figures and Tables

**Table 1:** Bacterial strains and plasmids used in this study.

Strains and plasmids	Description	Source
*Lactobacillus brevis* DT24	Probiotic vaginal isolate	In this study
*Escherichia coli* NCTC 50133	Contains pColE2-P9, produces colicin E2	[50]
*Escherichia coli* DH5*α*	Transformation host	MTCC
*Escherichia coli* BL21 D3	Expression Host	MTCC
*Escherichia coli *	Uropathogenic strain	MTCC

Plasmids	Description	Source

pSLP111.3	Cm^r^, *E. coli*/LAB shuttle vector *Slp*A signal peptide	Prof. Jos Seeger Lactrys, The Netherlands
pColE2-P9	Stp^r^, vector for colicin E2	*E. coli* Genetic Stock Centre, Yale University
pSL-ColE2	Cm^r^, *Slp*A, and ColE2	In this study
